# The Chest Pain That Never Went Away: A Case of Complex Cardiopulmonary Pathologies in a 64-Year-Old Caucasian Male

**DOI:** 10.7759/cureus.64746

**Published:** 2024-07-17

**Authors:** Muhammad Bilal, Muhammad H Malik, Ali Z Ansari, Ghazwan Bahro, Abhishek Jaiswal

**Affiliations:** 1 Department of Internal Medicine, Merit Health Wesley, Hattiesburg, USA; 2 Department of Pathology, William Carey University College of Osteopathic Medicine, Hattiesburg, USA; 3 Department of Interventional Cardiology, Merit Health Wesley, Hattiesburg, USA

**Keywords:** left heart catheterization, thoracentesis, ischemic cardiomyopathy, mass-like opacity, bilateral pleural effusion, hyperexpansion of lungs, interstitial coarsening, anginal chest pain, percutaneous coronary intervention (pci), st-segment elevation myocardial infarction (stemi)

## Abstract

Chest pain is a common and complex symptom that can arise from various etiologies, ranging from benign musculoskeletal conditions to life-threatening cardiovascular events. It is a hallmark symptom of myocardial infarction, angina, and other ischemic heart diseases, necessitating prompt and thorough evaluation. Ongoing chest pain post-procedures and medication administration presents a diagnostic challenge, as it may be indicative of an exacerbation of underlying conditions. We present the case of a 64-year-old Caucasian male who initially presented with severe and persistent chest pain suggestive of an anterior wall ST-elevation myocardial infarction (STEMI). He had a history of coronary artery disease and had recently undergone cardiac catheterization. Despite prompt administration of nitroglycerin and aspirin, the patient's symptoms persisted, prompting emergent percutaneous coronary intervention (PCI). Subsequent to PCI, ongoing chest discomfort persisted, prompting further investigation, which revealed a concurrent lung mass and nodules on imaging. Additional interventions, including repeated PCI procedures and thoracentesis, were undertaken. Unfortunately, the patient's clinical course rapidly deteriorated, culminating in cardiac arrest and unsuccessful resuscitative efforts. This case highlights the complexities inherent in managing intricate cardiovascular conditions and emphasizes the critical importance of maintaining vigilance for concomitant pathologies.

## Introduction

Chest pain is a prevalent and complex symptom that can indicate a variety of medical conditions. It is typically characterized by discomfort or pain in the chest area, which can vary in intensity and quality and may radiate to the arms, neck, jaw, or back. Ongoing chest pain following procedures and medications poses a diagnostic challenge, as it may stem from procedural complications, inadequate treatment response, or exacerbation of underlying conditions. Among these potential causes, acute coronary syndromes (ACS) stand out as a significant contributor, representing a spectrum of ischemic heart diseases that remain a leading cause of global morbidity and mortality [1]. Within this spectrum, ST-elevation myocardial infarction (STEMI) emerges as a critical subtype, characterized by persistent ST-segment elevation on electrocardiography (EKG) and indicative of complete occlusion of a coronary artery [2]. Prompt recognition and management of STEMI are paramount to mitigate myocardial damage and improve patient outcomes.

Despite advancements in reperfusion strategies, such as primary percutaneous coronary intervention (PCI) and thrombolytic therapy, STEMI continues to present formidable challenges due to its potential for rapid progression to cardiogenic shock, ventricular arrhythmias, and sudden cardiac death [3]. The multifaceted pathogenesis of STEMI, primarily rooted in atherosclerosis, underscores the importance of addressing modifiable risk factors, including hypertension, hyperlipidemia, and smoking [4]. However, the evaluation of ongoing chest pain extends beyond cardiac considerations to encompass a broad differential diagnosis, inclusive of pulmonary, gastrointestinal, musculoskeletal, and psychiatric etiologies. Gastroesophageal reflux disease (GERD), musculoskeletal strains, anxiety disorders, pneumonia, pleurisy, and pulmonary embolism (PE) represent a diverse array of conditions that can manifest with chest discomfort, necessitating a comprehensive clinical evaluation to discern the underlying pathology [5-7].

## Case presentation

**Figure 1:**
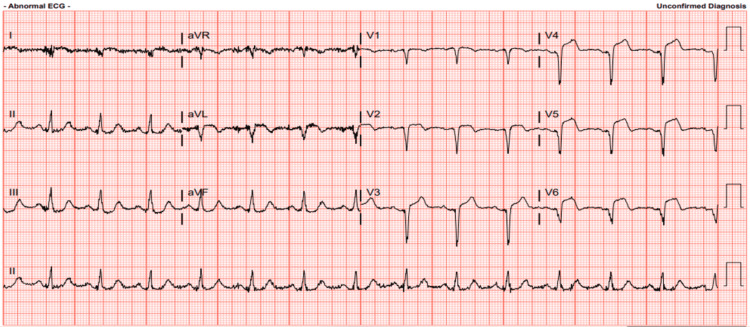
EKG revealing ST elevation indicative of anterolateral infarction during patient transport by EMS en route to the hospital. EKG: Electrocardiogram; EMS: Emergency medical services

**Figure 2:**
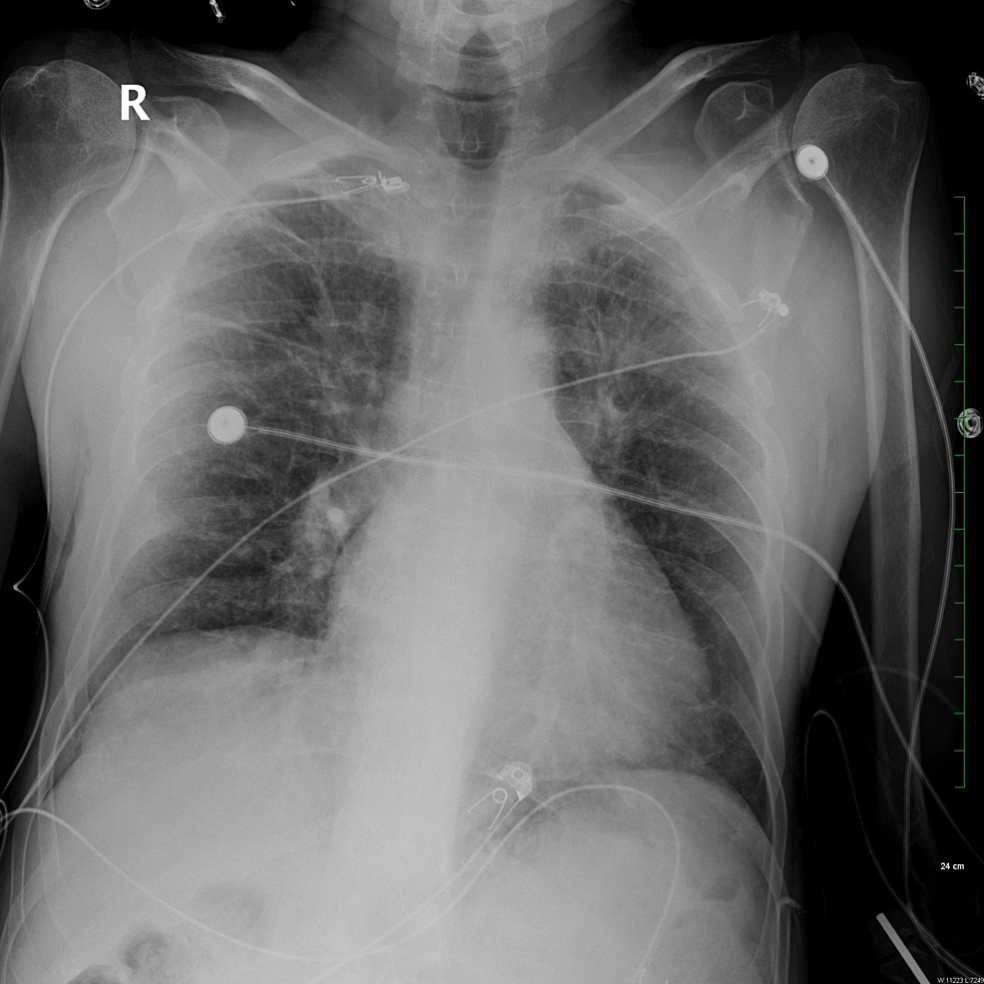
Chest X-ray revealing interstitial coarsening in the upper lung fields, accompanied by mild hyperexpansion upon hospital admission.

A 64-year-old Caucasian male presented to the Emergency Department (ED) with severe, persistent chest pain that began abruptly shortly before his arrival. Emergency medical services (EMS) found him lying on his porch and transported him to the hospital. His medical history includes a recent cardiac catheterization revealing coronary artery disease. On initial assessment, the patient denied any accompanying symptoms and reported no factors that alleviated or worsened the chest pain. His vital signs showed a blood pressure of 188/115 mmHg. Prior to admission, EMS had administered aspirin and nitroglycerin, and their records noted ST elevation on the EKG (Figure 1). A chest X-ray revealed a normal heart size and vascularity but showed interstitial coarsening in the upper lung fields and mild hyperexpansion (Figure 2). Initial laboratory investigations revealed elevated troponin levels at 129.8 ng/mL. To address his discomfort, the patient received morphine, ondansetron (Zofran), and intravenous heparin.

Following initial assessment, the patient underwent emergent left heart catheterization (LHC), which revealed significant involvement of the proximal to mid-left anterior descending (LAD) artery and obtuse marginal branch. Successful revascularization of the proximal LAD was accomplished using a 3.5 x 28 mm Xience drug-eluting stent (Abbott Laboratories, Abbott Park, IL, USA), followed by post-dilation with a 4.0 x 15 mm noncompliant balloon, resulting in a remarkable lesion reduction from 100% to 0%. Subsequently, the patient was commenced on a therapeutic regimen comprising ticagrelor (Brilinta) and aspirin. Despite the intervention, the patient persisted in reporting chest pain and soreness in the mid-chest region post-catheterization. Notably, the cardiologist had previously recommended LHC one month prior to the current presentation, which the patient had declined at that time.

**Figure 3:**
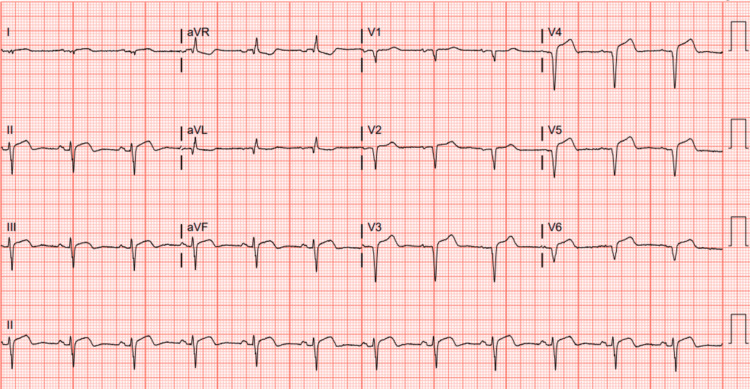
EKG showing sustained ST elevation raising concerns for left ventricular aneurysm the morning after LHC. EKG: Electrocardiogram; LHC: Left heart catheterization

The morning following catheterization, the patient experienced a dramatic surge in troponin levels, peaking above 125,000 ng/mL before gradually declining to 89,055 ng/mL and further reducing to 41,000 ng/mL over subsequent days. Despite this, the patient continued to report persistent chest pain in the days following the procedure. A repeat EKG conducted during this time exhibited sustained ST elevation consistent with previous recordings, raising concerns about the potential development of a left ventricular (LV) aneurysm (Figure 3). Concurrently, an echocardiogram revealed an ejection fraction (EF) of 41% with segmental wall abnormalities, while ventricular size remained within normal parameters. On the fourth day of hospitalization, an incident involving a recent fall and subsequent chest pain prompted medical attention. Nitroglycerin was administered, and despite a blood pressure reading of 96/61 mmHg, a repeat EKG did not reveal any new pathological findings.

**Figure 4:**
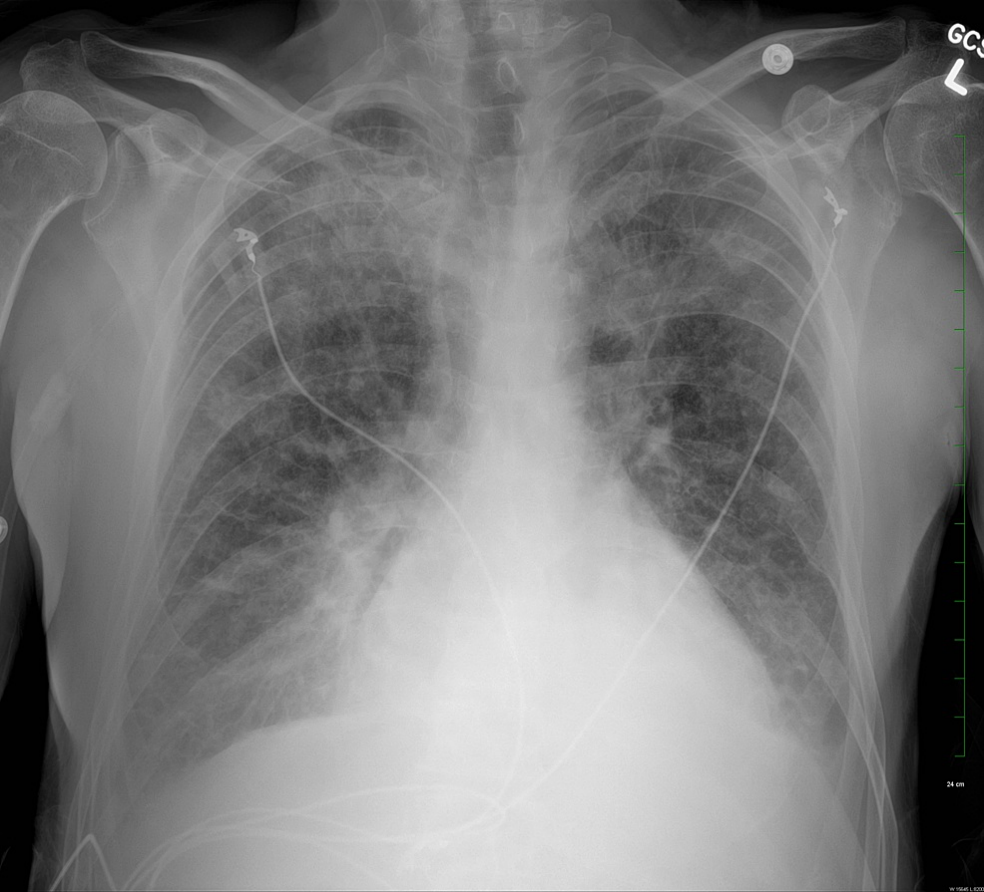
Chest X-ray revealing mild exacerbation of airspace opacities and bilateral pleural effusion during the seventh day of hospitalization.

**Figure 5:**
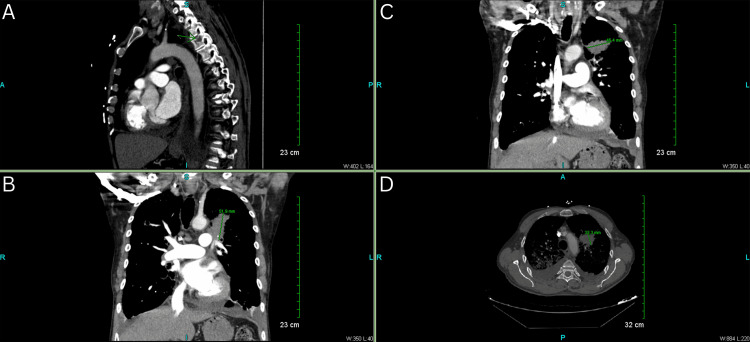
CT chest findings demonstrating lytic lesions involving the right seventh rib, left ninth rib, and T4 vertebral body (A). Additionally, a mass-like opacity with spiculated margins in the left upper lobe perihilar region is observed, measuring 5.2 cm craniocaudally (B), 4.6 cm transversely (C), and 3.3 cm anteroposteriorly (D). Multifocal pulmonary consolidation suggestive of pneumonia and bilateral pleural effusions are also observed. CT: Computed tomography

In the subsequent days, although the patient's troponin levels exhibited a declining trend, the persistence of burning chest pain remained a concern. A repeat chest X-ray performed on the seventh day of hospitalization revealed a subtle exacerbation of airspace opacities, alongside minor bilateral pleural effusion (Figure 4). Considering the patient's immobilized state and ongoing chest discomfort, a D-dimer assay was conducted, yielding a result of 756 ng/mL and prompting consideration of a PE. Consequently, a contrast-enhanced computed tomography angiography (CTA) of the chest was arranged on the same day, revealing the absence of acute PE. However, the CTA identified a mass-like opacity with spiculated margins in the left upper lobe perihilar region, measuring 5.2 cm in craniocaudal dimension, 4.6 cm in transverse dimension, and 3.3 cm in anteroposterior dimension (Figure 5). This mass encroached upon the pulmonary arteries to the left upper lobe and extended into the mediastinal fat, accompanied by mild mediastinal and right hilar lymphadenopathy. Additionally, bilateral pleural effusions and atelectatic changes were noted, with the mass abutting the aortic arch.

**Figure 6:**
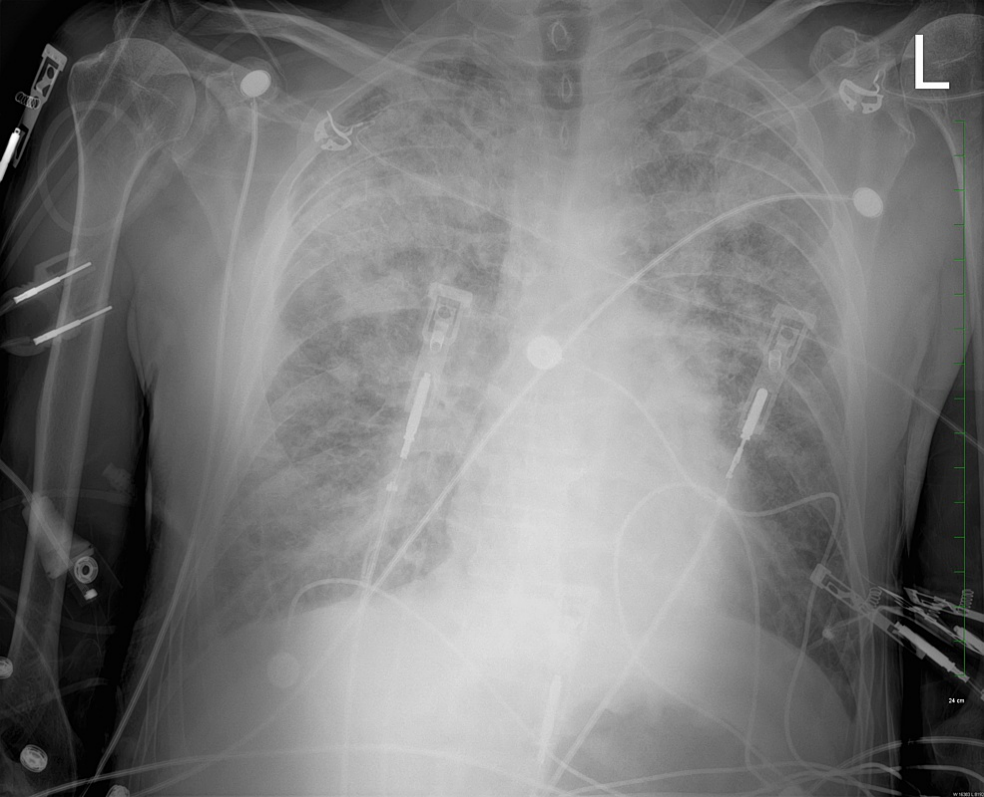
Chest X-ray reveals marked exacerbation of airspace opacification bilaterally, notably in the upper lobes during the ninth day of hospitalization.

Consultation with the Medicine team was sought, leading to the decision for an interventional radiology (IR)-guided biopsy. Subsequently, the Pulmonology team determined a 99% probability of malignancy associated with the detected nodule. However, the biopsy procedure was deferred due to the patient's ongoing anticoagulant therapy. A repeat chest X-ray on the ninth day of hospitalization, prompted by worsening crackles upon auscultation, revealed a significant exacerbation of lung opacification (Figure 6). On the same day, a rapid response was initiated due to the patient's severe chest pain, culminating in the identification of EKG findings consistent with STEMI. Consequently, the patient underwent a second LHC. During this procedure, no new lesions or stent thrombosis were identified. The revascularization involved the placement of an additional stent in the previously treated LAD artery to ensure complete vessel patency. Post-procedurally, the patient was prescribed aspirin and ticagrelor (Brilinta).

The repeat LHC revealed ischemic cardiomyopathy alongside moderate LV systolic dysfunction. Notably, thrombus formation was detected within the body of the LAD stent, despite thrombolysis in myocardial infarction-3 (TIMI-3) flow. The likely mechanism for thrombus formation included suboptimal stent expansion or inadequate dual antiplatelet therapy efficacy. Additionally, intravascular ultrasound revealed stenosis in the proximal LAD and left main coronary artery in the native vessels. The stenosis was successfully treated with balloon angioplasty using a 3.0 x 15 mm noncompliant balloon and stent placement in the obtuse marginal branch, without complications. The patient's medication regimen of ticagrelor (Brilinta) and aspirin was continued.

During the procedure, an LV angiogram was performed, confirming the diagnosis of moderate LV systolic dysfunction. Following the second catheterization, the patient was transferred to the coronary care unit (CCU). Concurrently, a thoracentesis of the left pleural space was performed, yielding 750 mL of straw-colored fluid. Subsequent to the thoracentesis, the patient experienced improved breathing and alleviation of shortness of breath.

Three days later, the patient's clinical condition showed significant improvement, leading to a step-down from the intensive care unit (ICU) as vasopressor support became unnecessary and oxygen requirements decreased. However, shortly thereafter, the patient experienced a sudden deterioration in neurological status, characterized by altered mental status and complaints of chest pain preceding a cardiac arrest. Subsequently, the patient became apneic, necessitating emergent endotracheal intubation by the ED team. Despite resuscitative efforts, including cardiopulmonary resuscitation (CPR), the patient could not be revived and was declared deceased.

## Discussion

This case of a 64-year-old Caucasian male with complex cardiopulmonary pathologies demonstrates the diagnostic challenges encountered in clinical practice. Initially presenting with symptoms highly suggestive of an anterior wall STEMI, the patient's management pathway was directed towards emergent revascularization via PCI. Despite the successful intervention, characterized by significant lesion reduction in the LAD, the persistence of chest discomfort post-procedure raised concerns regarding ongoing myocardial ischemia.

In post-PCI patients, the persistence of chest pain can be multifactorial, encompassing a spectrum of cardiac and non-cardiac etiologies. While immediate post-procedural chest discomfort is commonly attributed to vascular injury and inflammation, prolonged or recurrent symptoms mandate a comprehensive evaluation to discern the underlying pathology [8]. In this case, the decline in troponin levels following PCI argued against ongoing ACS as the sole explanation for the patient's symptoms. However, the emergence of sustained ST elevation on repeat EKG, reminiscent of potential complications such as LV aneurysm, warranted heightened surveillance.

The presence of a lung mass and nodules introduces several potential mechanisms by which pulmonary pathology may influence cardiovascular function and precipitate adverse cardiac events. Firstly, the mass itself can exert mechanical compression on adjacent structures, including the heart and major vessels, leading to hemodynamic compromise [9]. Depending on the location and size of the mass, compression of pulmonary arteries or adjacent cardiac structures could impede blood flow, exacerbating underlying ischemic cardiac conditions and predisposing the patient to arrhythmias or cardiac failure [10].

Moreover, the inflammatory response elicited by the lung mass can contribute to systemic inflammation and endothelial dysfunction, further exacerbating cardiovascular pathology. Tumor-associated cytokines and inflammatory mediators released by the malignant cells can promote a prothrombotic state, endothelial activation, and vascular remodeling - all of which predispose to thromboembolic events and coronary artery thrombosis [11]. Additionally, the release of vasoactive substances, such as endothelin and prostaglandins, can induce vasoconstriction and impair myocardial perfusion, exacerbating ischemic heart disease [12].

Furthermore, the presence of pulmonary nodules raises concerns for metastatic disease, with the potential for hematogenous spread to the heart and systemic circulation. Metastases to the heart can lead to intracardiac obstruction, valvular dysfunction, and arrhythmias, further compromising cardiac function and precipitating cardiac events [13]. The systemic effects of metastatic disease, including paraneoplastic syndromes and metabolic derangements, can also adversely impact cardiovascular function and contribute to the patient's clinical deterioration [14].

## Conclusions

This case illustrates the intricacy between cardiovascular and pulmonary pathologies, emphasizing the diagnostic and therapeutic challenges inherent in clinical practice. Despite prompt recognition and management of STEMI through emergent PCI, the persistence of chest discomfort necessitated further investigation, leading to the identification of a concurrent lung mass and nodules. Further research is warranted to optimize strategies for the management of patients with concomitant cardiopulmonary pathologies.
